# Editorial: Personalized nutrition in chronic kidney disease

**DOI:** 10.3389/fnut.2024.1422149

**Published:** 2024-05-30

**Authors:** Lara Caldiroli, Andreana De Mauri, Alice Sabatino

**Affiliations:** ^1^Department of Nephrology, Dialysis and Kidney Transplants, IRCCS Ca 'Granda Foundation Maggiore Policlinico Hospital, Milan, Italy; ^2^Nephrology and Dialysis Unit, Maggiore della Carità University Hospital, Novara, Italy; ^3^Division of Renal Medicine, Baxter Novum, Department of Clinical Science, Intervention and Technology. Karolinska Institute, Stockholm, Sweden

**Keywords:** chronic kidney disease, nutritional assessment, low protein diet, renal nutrition and metabolism, dialysis, kidney transplantation

Nutritional status is an important prognostic factor in patients with chronic kidney disease (CKD). The nutritional approach depends on the stage of kidney disease, patient comorbidities, protein catabolism and the presence of malnutrition.

Close nutritional follow-up, including regular nutritional assessment and supervised dietary modifications, is key to the management of CKD (1, 2), and plays an important role in controlling the signs, symptoms and metabolic complications of CKD, while preventing and treating malnutrition and protein energy wasting (PEW). Several dietary approaches have been proposed but the most successful nutritional regimen is the one tailored on patients' needs (3).

The latest K-DOQI guidelines for dietary management of CKD extend the range of dietary interventions, particularly protein restriction, to stage 3 CKD regardless of age and highlight that optimisation of protein intake is associated with a reduction in mortality and morbidity (4). However, due to the accumulation of uremic toxins, there is a tendency for spontaneous reductions in protein and energy intake in older patients with CKD (5–8), which may compromise overall nutritional status, if patients are not monitores and nutritional counseling is not present. Because of these risks, some clinics tend to limit nutritional modification in the diet of older patients with CKD, leading to heterogeneity between centers and in a larger scale, between countries (9). In this Research Topic of Frontiers in Nutrition, we have interesting papers on different areas of nutritional management of CKD. Vettoretti et al.'s study sheds light on the prevalence of spontaneous low-protein intake in older patients with CKD. The authors found that among older patients attending a CKD outpatient clinic, almost two-thirds had a spontaneous reduction in dietary protein intake and they were more frail, had worse nutritional status and physical performance in comparison to patients that did not presented a spontaneous reduction in protein intake. These findings highlight the importance of assessing patients' needs and personalized approaches with individual risk-benefit assessments. To achieve the best possible outcomes, targeted interventions should use all available tools. Valuable insights into the validity and applicability of the Global Leadership Initiative on Malnutrition (GLIM) criteria in non-dialysis CKD patients are provided by the study by Huang et al. By comparing different screening tools and indicators, the authors highlight the importance of incorporating the assessment of reduced muscle mass into the GLIM framework to improve diagnostic accuracy. They also highlight the potential of adding handgrip strength measurements to increase sensitivity in identifying cases of malnutrition. In addition, Pu et al.'s work addresses the need for standardized nursing terminology in CKD nutrition management to facilitate comprehensive care and data sharing. By establishing a subset of standardized nursing terminologies, the study paves the way for improved communication and collaboration among healthcare providers in managing the nutritional needs of CKD patients.

Many factors are involved in the deterioration of nutritional status in patients with CKD. These include unsupervised dietary changes which, combined with the loss of appetite often observed in this patient population, may lead to spontaneous reductions in energy and nutrient intake (10). In addition, negative energy and protein balance in patients with CKD/ESKD can be caused by the catabolic effects of kidney replacement therapy (KRT), metabolic and hormonal derangements, the presence of systemic inflammation and comorbidities, and reduced physical activity (10, 11).

The clinical study by Tao et al. adds to the growing body of evidence supporting the efficacy of personalized nutrition therapy in ESKD patients on dialysis. This study analyzed changes in residual renal function (RRF) and indicators of blood and kidney function in ESKD with personalized nutritional therapy. The results show that nutritional interventions for ESKD are effective in reducing the rate of decline in RRF and improving indicators of blood and kidney function in patients on dialysis. Their findings highlight the potential of nutritional interventions to preserve RRF and improve overall clinical outcomes in this population. Additionaly, a systematic review and meta-analysis by Ren et al. provides compelling evidence for the efficacy of oral nutritional supplements (ONS) in dialysis patients. By synthesizing data from randomized controlled trials (RCTs), the study highlights the beneficial effects of ONS on key nutritional parameters, including body mass index (BMI), serum albumin levels, nitrogen balance and markers of systemic inflammation. These findings underscore the importance of including ONS as part of a comprehensive nutritional programme for dialysis patients, providing clinicians with a valuable tool to optimize patient care. However, as Ocepek et al.'s interventional study suggests, simply adding ONS to a regular diet may not be sufficient to address the multiple challenges of protein energy wasting in haemodialysis patients. The study highlights the need for comprehensive nutritional approaches tailored to individual patient needs, including personalized dietary counseling, close monitoring of nutritional parameters during dialysis treatment and psychological support.

Changing the perspective prevention, some studies have shown that there is an association between diet-induced inflammation and the presence of common chronic diseases (12). However, there has been uncertainty about the influence of dietary inflammatory potential on the risk of chronic kidney disease (CKD) in middle-aged and older groups. On this regard, Guo et al.'s research looked at the relationship between the Dietary Inflammatory Index (DII) and CKD in people aged 40 years and older, showing a positive association between DII and CKD in this age group.

Dietary patterns have been shown to be closely related to inflammation, which has been independently associated with cognitive impairment (CI) in patients undergoing haemodialysis (HD). However, the influence of inflammation-derived dietary patterns on CI in this population remains unclear. Zhuang et al.'s investigation of the relationship between inflammation-derived dietary patterns and cognitive impairment in haemodialysis patients sheds light on the complex interplay between diet, inflammation, and cognitive health in this population. Dietary patterns that were associated with high CRP levels included high intake of rice, tea and coffee, alcohol and fruits, and low intake of dark vegetables and juice, and they contributed to an increased risk of CCI. The association between consumption of seafood, sweet drinks, and alcohol and CCI has not yet been established. However, they may be dietary factors that contribute to inflammation in patients undergoing HD.

Another important challenge in the current nutritional management of patients with ESKD on haemodialysis is achieving a good dietary quality pattern (13). Fear of hyperkalemia often prevents clinicians from encouraging patients to eat fruits and vegetables, resulting in poor dietary fiber intake. A low dietary fiber intake seems to be associated with the development of low-grade systemic inflammation by promoting intestinal dysbiosis and should not be overlooked (14, 15). Bi et al.'s longitudinal observational study explores the complex relationship between gut microbiota dysbiosis and protein energy wasting (PEW) in haemodialysis patients. By examining changes in gut microbiota composition over time, the study identifies potential microbial markers associated with muscle mass loss and the occurrence of PEW. Such findings hold promise for the development of targeted interventions aimed at modulating the gut microbiota to reduce the risk of PEW in dialysis patients.

Rounding off this Research Topic, Zhang et al. describes the relationship between immune-nutrition indices and mortality outcomes in patients with CKD. The Mendelian randomization study by Yin et al. provides valuable insights into the causal relationships between serum metabolites and CKD incidence and progression. Li et al. evaluates the role of specific amino acid metabolism in the development of diabetic kidney disease (DKD), providing new insights into the pathophysiological mechanisms underlying disease progression. Finally, Song et al. investigates the association between coffee consumption and serum uric acid levels in a US population with CKD.

Taken together, these studies underscore the multifaceted nature of nutritional care in CKD patients and highlight the importance of personalized interventions tailored to individual needs. We are confident that this reading will provide new information for clinicians and researchers in the field of nutrition and kidney disease.

## Author contributions

LC: Writing—original draft. AD: Writing—review & editing. AS: Writing—review & editing.
